# Repair of giant subcostal hernia using porcine acellular dermal matrix (Strattice™) with bone anchors and pedicled omental flap coverage: a case report

**DOI:** 10.1186/1752-1947-7-258

**Published:** 2013-11-11

**Authors:** Jonathan King, J David Hayes, Bryan Richmond

**Affiliations:** 1 West Virginia University School of Medicine, 1 Medical Center Drive, Morgantown, WV 26506, USA; 2 Charleston Area Medical Center Plastic Surgery Center, 501 Morris Street, Charleston, WV 25301, USA; 3 Department of Surgery, West Virginia University/Charleston Division, 3110 MacCorkle Avenue SE, Charleston, WV 25304, USA

**Keywords:** Subcostal, Hernia, Omental flap, Porcine acellular dermal matrix, Strattice

## Abstract

**Introduction:**

Giant abdominal wall hernias represent a major challenge to the hernia surgeon in practice today. Of the common abdominal wall hernias, those located in the subcostal region are among the most difficult to repair, and have historically been plagued by higher recurrence rates than other locations, such as the midline. No technique has been identified as the clearly superior choice for hernias of this type.

**Case presentation:**

We report a successful repair of a giant, multiply recurrent subcostal hernia with loss of domain in a 45-year-old obese Caucasian man. This was accomplished in a novel fashion, using a porcine acellular dermal matrix (Strattice™) as the floor of the repair, which was fixed to the costal margin using orthopedic bone anchors (Mitek™), then covered with a pedicled omental flap to eliminate dead space and facilitate a more rapid revascularization of the porcine acellular dermal matrix implant.

**Conclusions:**

This case emphasizes the need for a thorough understanding of the challenges of the specific type of hernia defect encountered, as well as knowledge of any available techniques that may be adjunctively employed to enhance the chances of achieving a successful result.

## Introduction

Incisional hernias remain a frequent complication of abdominal surgery and result in approximately 250,000 ventral hernia repairs in the United States annually [1]. Repair of complex ventral hernias poses a major challenge to abdominal wall surgeons, and the repair of such defects has historically been plagued by both high recurrence rates and significant associated morbidity. Many techniques for repair have been described, including primary suture repair, synthetic mesh repair (both open and laparoscopic), repair with biologic tissue matrices, and component separation [2-4], both with and without reinforcement with biologic or synthetic materials. The majority of these techniques and the results achieved have been reported in repair of midline ventral incisional hernia defects.

Repair of non-midline ventral hernia poses an even greater challenge to surgeons for many reasons. While management options for midline hernia repair are well known, guidelines for management of lateral abdominal wall hernia repair are lacking. This is especially true of hernia in the subcostal location, which has been demonstrated to have the highest recurrence rate among non-midline hernias, although the exact incidence of failure is not well described in the literature [5].

## Case presentation

A 45-year-old Caucasian man was referred for evaluation of a large right subcostal hernia and associated loss of abdominal domain, in that the majority of the patient’s abdominal contents were outside the abdominal cavity. Three years prior to referral he had undergone an open cholecystectomy via a generous right subcostal incision. He subsequently developed a lateral subcostal incisional hernia, which was repaired on three separate occasions using an underlay of composite synthetic mesh. The third repair was complicated by infection of the mesh, which required explantation of the prosthesis. The skin was simply closed over the remaining hernia defect, which continued to enlarge, resulting in loss of domain and ulceration of the overlying skin. Fearing evisceration, the patient sought a second opinion at our institution.

**Figure 1:**
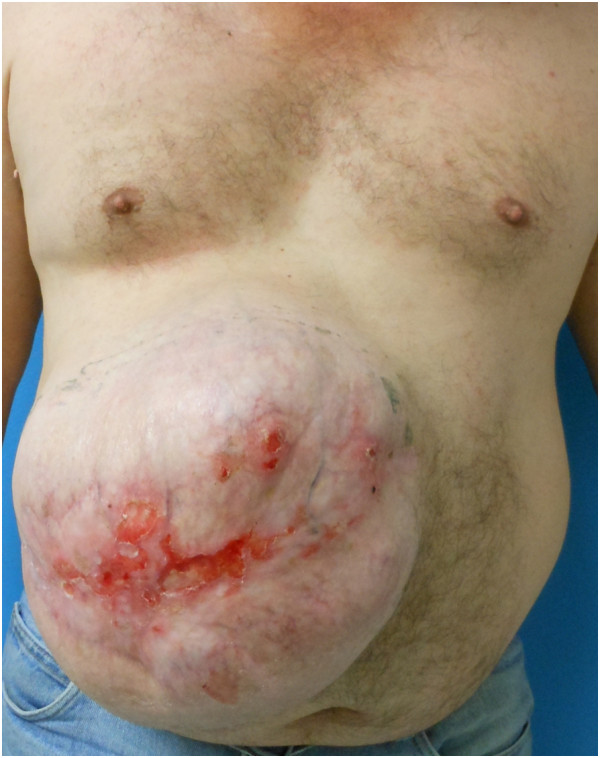
Preoperative anterior view of large right upper quadrant incisional hernia with focal areas of skin ulceration.

On initial presentation, our patient was noted to be obese (with a body mass index of 33.6kg/m^2^). He acknowledged tobacco usage of one pack per day. A history of diabetes mellitus was not present. On examination, he was noted to have an extensive subcostal hernia defect, with the fascial defect measuring approximately 25 × 20cm (500cm^2^) by palpation, with ulceration of the overlying skin (Figure 1). The patient was counseled to stop smoking, which he subsequently did. He was reevaluated approximately eight weeks later, at which time he was scheduled for elective repair.

**Figure 2:**
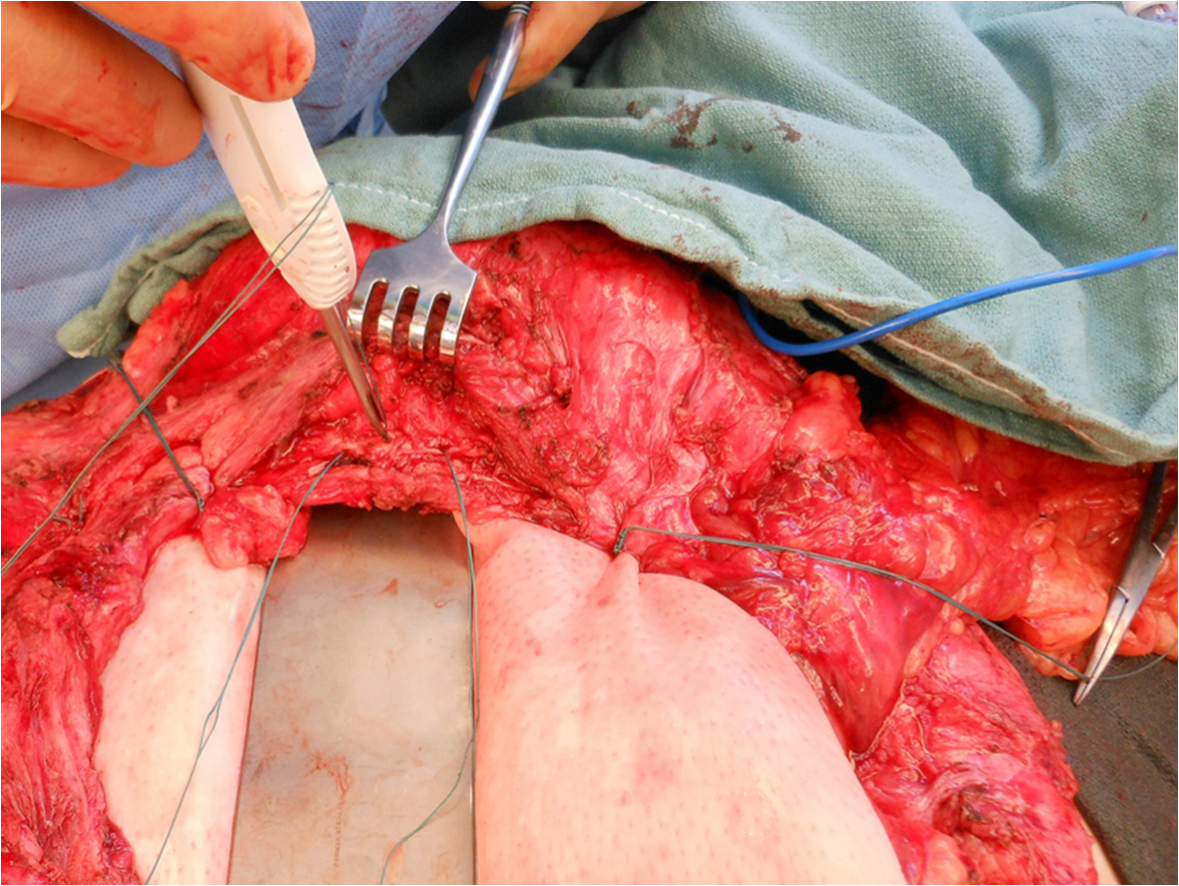
Fixation of the porcine-derived acellular dermal matrix to the body of rib using Mitek™ bone anchors.

**Figure 3:**
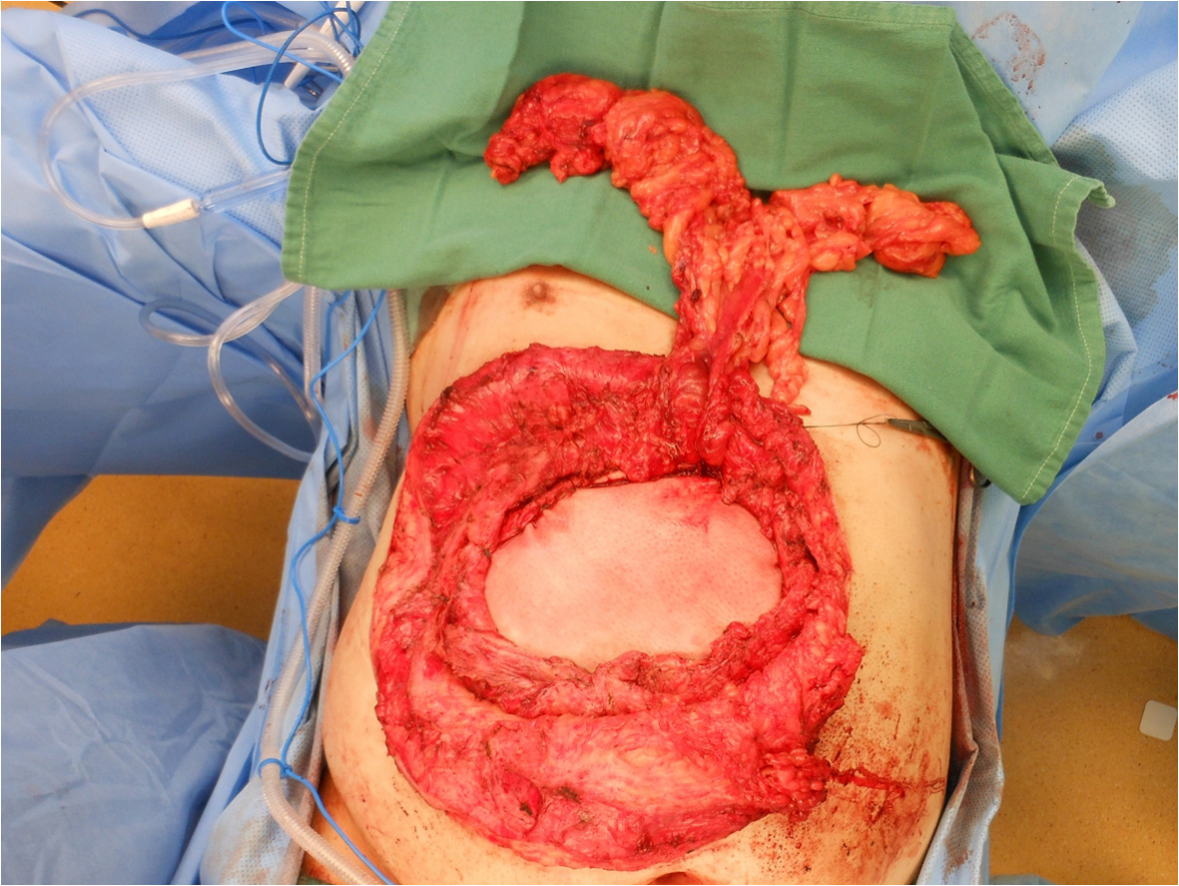
Intraoperative view of the porcine-derived acellular dermal matrix sutured in place in preparation for coverage with a raised pedicled omental flap.

**Figure 4:**
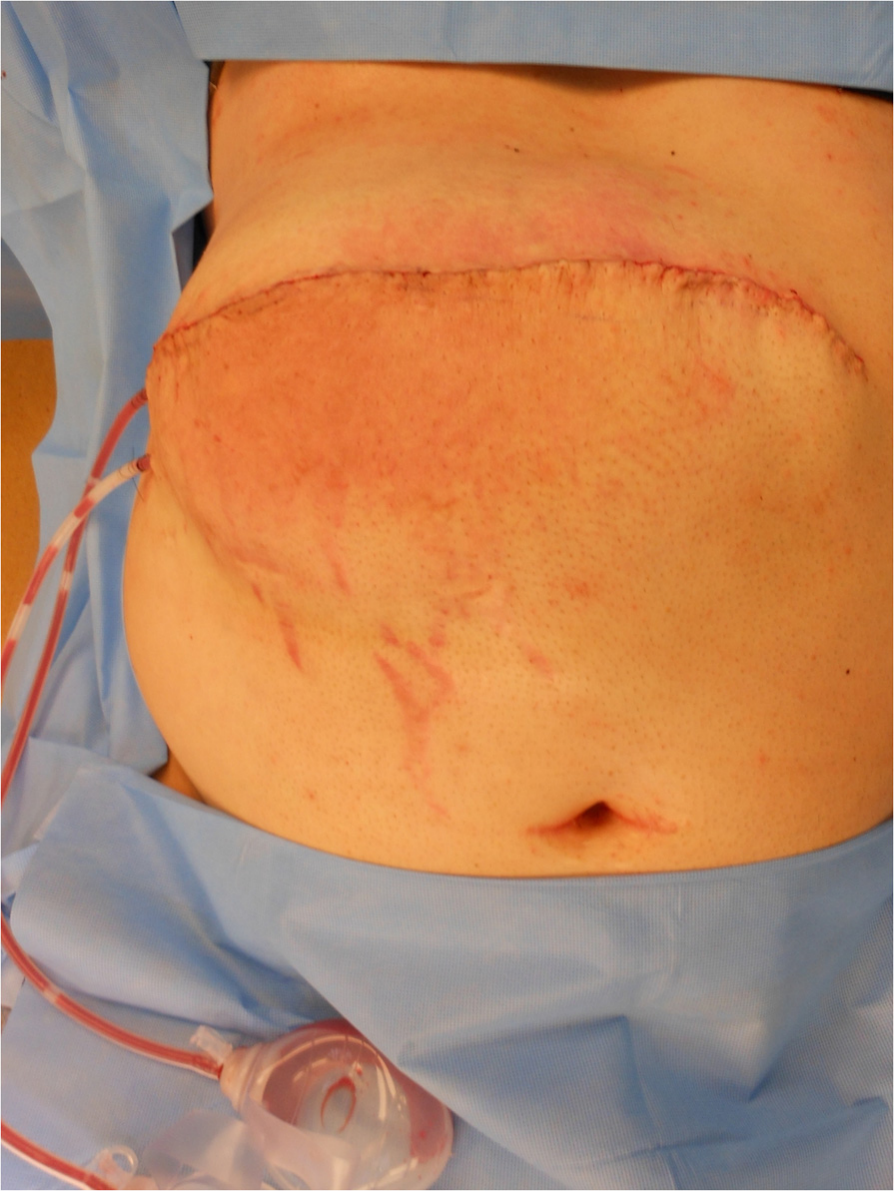
Postoperative view of the repair.

Repair of the hernia was approached as follows: The ulcerated skin overlying the defect was excised completely via an elliptical incision. An extensive enterolysis was then performed to free the viscera from the overlying skin and the undersurface of the fascia. An omental flap measuring 25cm in length and 10cm in width was mobilized, based on the right gastroepiploic blood supply. Subcutaneous flaps were then raised to expose the inferior, medial, and lateral fascial surfaces for fixation of a porcine acellular dermal matrix (PADM, Strattice™, LifeCell Corporation, Branchburg, NJ, USA). PADM was chosen due to the presence of active skin ulceration. No fascia could be identified superiorly, so the superior subcutaneous flap was raised to expose the costal margin. A 20 × 30cm piece of PADM was placed into the wound and secured to the medial, lateral, and inferior aspects of the defect using interrupted permanent mattress sutures placed 2cm apart with a minimum of a 5cm underlay. Superiorly, the PADM was secured to the body of the rib using Mitek™ (DePuy, Raynham, MA, USA) bone anchors with pre-attached sutures, placed 2 to 3cm apart (Figure 2). After placement of the bone anchors, the suture material was passed over the superior border of the rib and into the abdomen, thereby avoiding the neurovascular bundle. The sutures were then used to achieve a 5cm underlay under the costal margin as well. A small defect measuring 2 × 3cm was left in the epigastric region immediately below the xiphoid process, which was used to deliver the omental flap into the wound (Figure 3). The flap was oriented over the PADM implant, two 19 French™ (Ethicon, Somerville, NJ, USA) closed suction drains were placed, and the skin flaps were closed over the repair (Figure 4).

**Figure 5:**
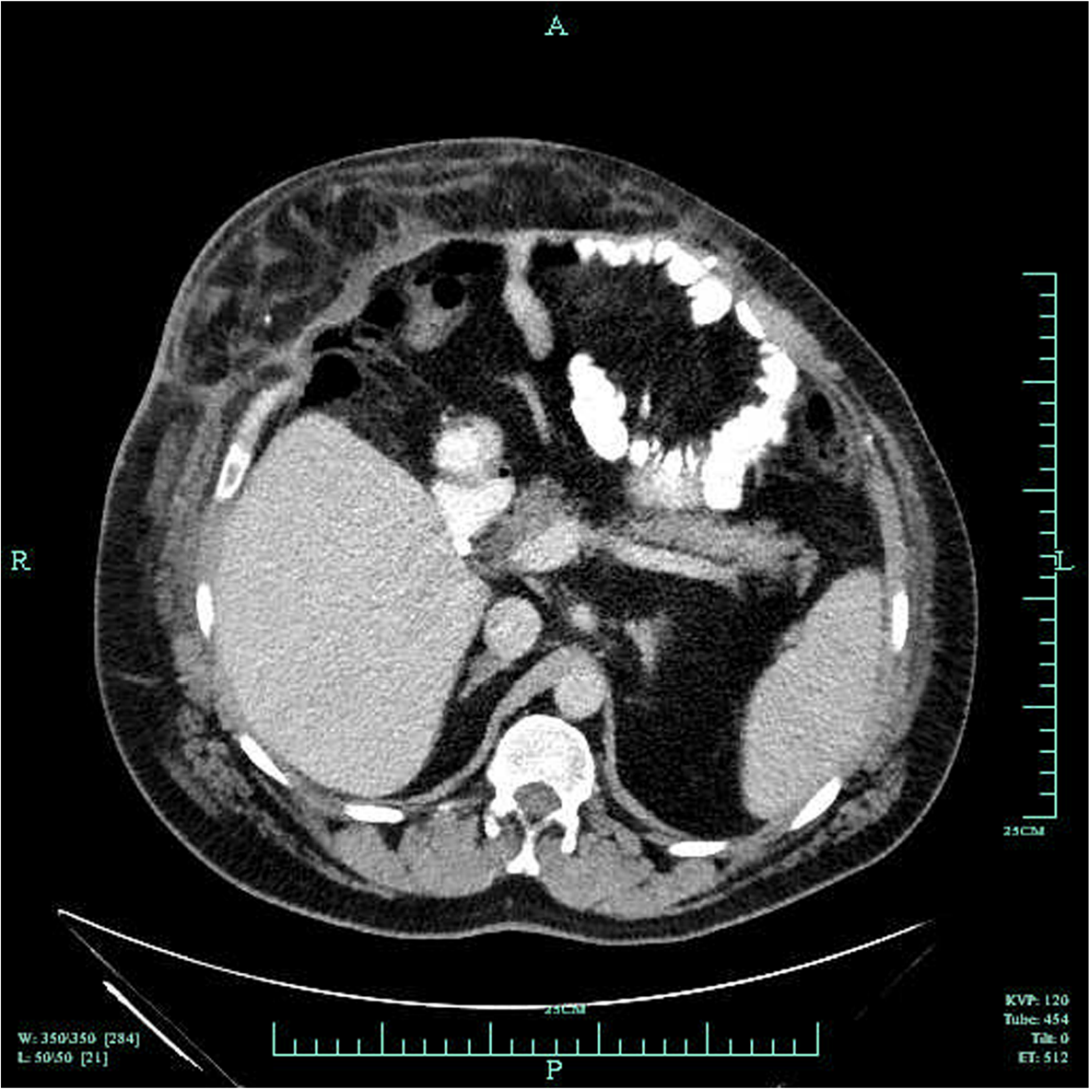
**Computed tomography scan of the abdomen showing the intact hernia repair.** Note the presence of the omental flap above the porcine-derived acellular dermal matrix.

Postoperatively, antibiotics were discontinued within 24 hours. The patient did well and was discharged home on postoperative day 5 with all drains removed. His wound healed without complication. Follow-up computerized tomography was done six weeks postoperatively, which revealed no evidence of recurrence or seroma (Figure 5).

**Figure 6:**
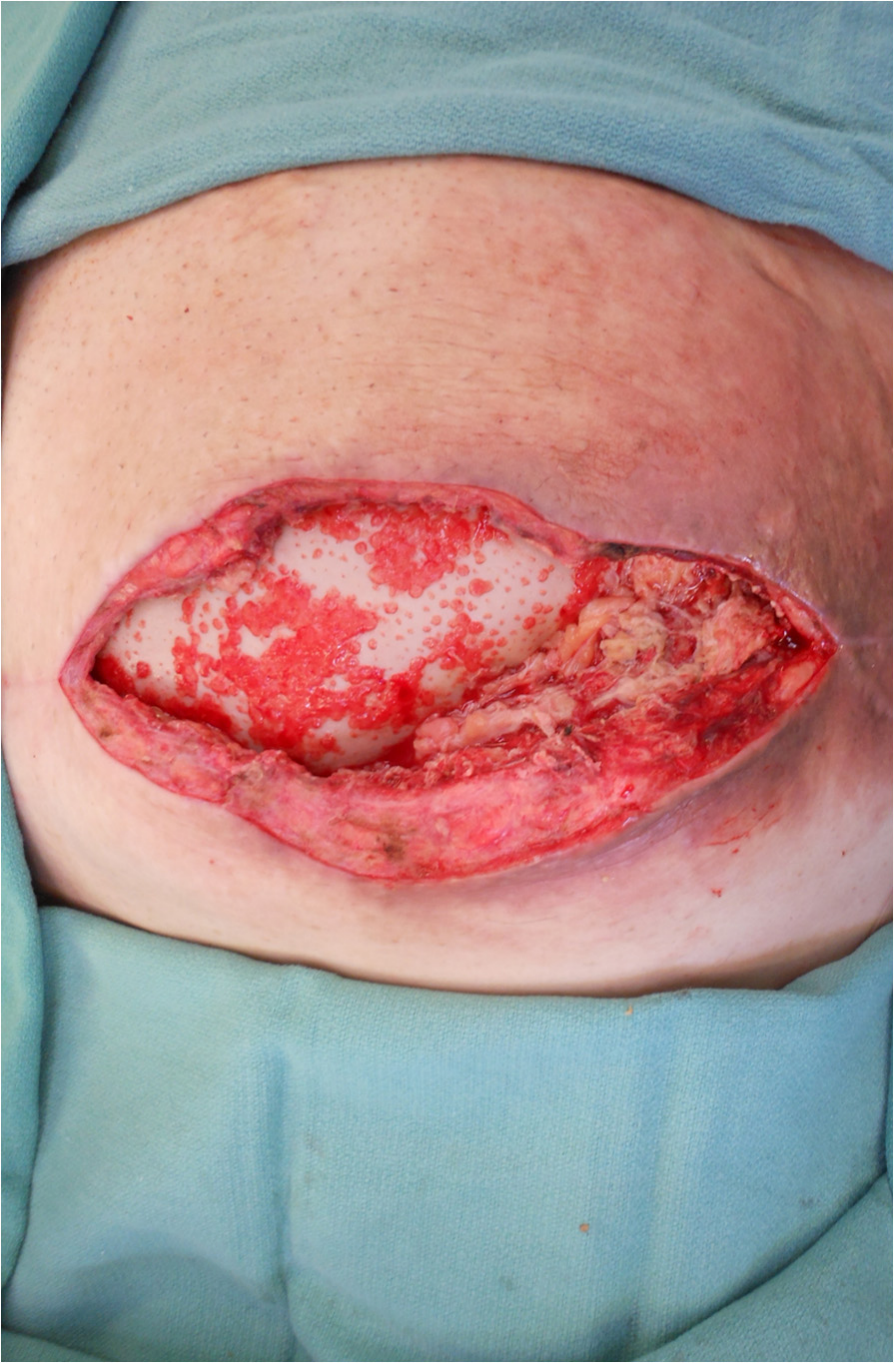
**Photograph of the exposed porcine-derived acellular dermal matrix after wound debridement.** Note exuberant granulation tissue indicating neovascularization of the implant.

**Figure 7:**
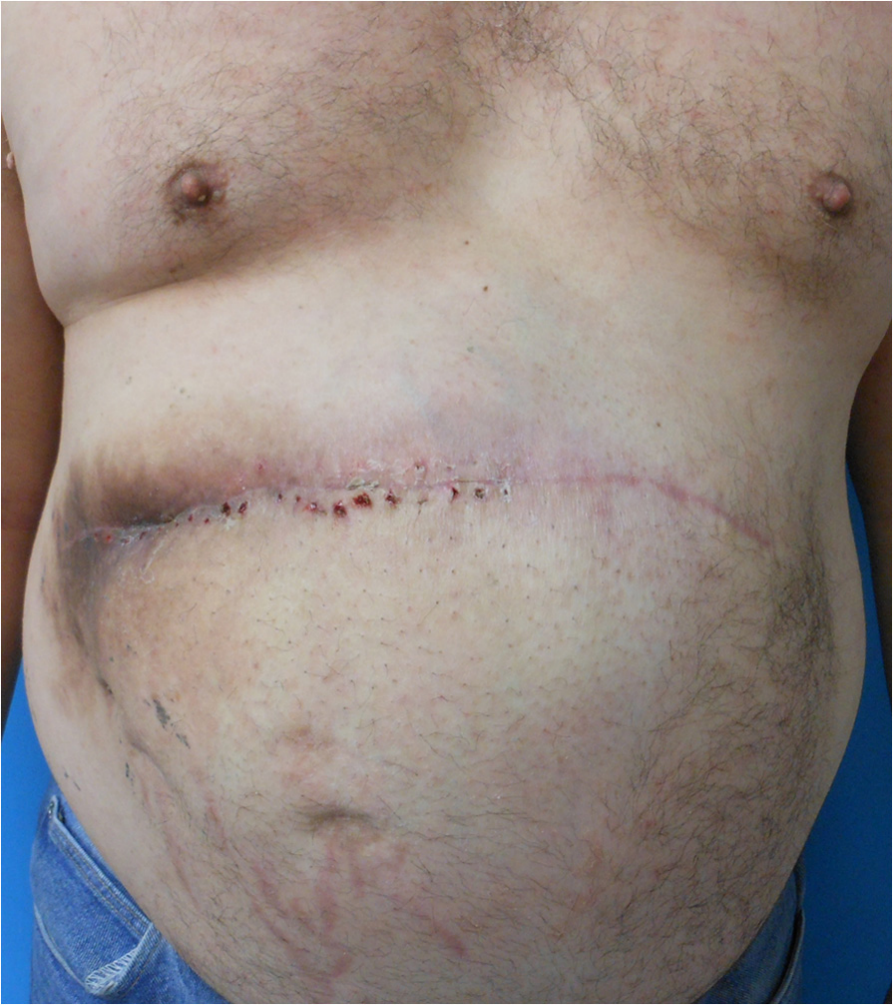
View at six-month follow-up after removal of the necrotic portion of the omental flap.

Approximately nine weeks postoperatively, he presented to the clinic with fever, as well as erythema and fluctuance of his incision site. He was subsequently taken to the operating room for wound exploration and debridement. Upon opening the wound, it was discovered that a portion of the omental flap had become necrotic. This was excised without difficulty and the remaining healthy omentum left *in situ*. The PADM underlay repair was still intact and was fully incorporated at the wound edges. Exuberant granulation tissue was present on the surface of the PADM, indicating ongoing neovascularization (Figure 6). The wound was then re-closed over drains and our patient was discharged the following day. He healed without further incident and has no evidence of recurrent hernia at six months of follow-up (Figure 7).

## Discussion

The ventral hernia literature published to date has primarily focused on the management of the more commonly encountered midline hernia defect. However, lateral abdominal wall hernias pose a unique challenge owing to their complexity and limited surgical options for repair [6]. The physiology is also quite different from that of a midline defect, which directly contributes to the difficulty in repairing lateral abdominal wall defects and the associated higher recurrence rates associated with repairs of defects of this type, although the exact failure rate is difficult to ascertain, owing to the inherent heterogeneity of the patient population. An imbalance exists in the distraction forces on lateral abdominal wall hernias (and any subsequent repairs) due to the off-center nature of the defects. Also, the ratio of muscle to more durable fascia is higher in the lateral abdominal wall, which contributes to the lower integrity of the tissues available for repair of lateral defects. Finally, it is often not possible to mobilize tissues locally to close the defect as can often be done with component separation in the management of midline hernias. This often results in the need for bridging of the defect as the necessary technique of repair. All of these factors contribute to the higher failure rate of lateral abdominal wall repairs when compared to results obtained with the repair of midline defects [6].

The anatomy involved in a subcostal hernia includes all layers of the lateral abdominal wall musculature – the external oblique, internal oblique, and transversus abdominis muscle. The neurovascular bundles of the lateral abdominal wall muscles are obliquely oriented and easily injured; if injury occurs, muscle denervation and further weakening of the tissue results. This may occur as a result of progressive enlargement of the defect, further complicating attempts at repair [7]. Additionally, each of these layers has relatively little aponeurotic substance when compared to that of the midline, providing fewer points of secure fixation for a successful hernia repair. This sometimes results in the need for the fixation of any reinforcing (or bridging) material to bony points of fixation such as the costal margin [6,7], which was necessary in our case. Various techniques for this have been described, including passing a suture around the rib circumferentially, the insertion of suture into the rib itself via pre-drilled holes [6], or the Mitek™ bone anchor fixation system [8]. The Mitek™ system functioned well in our case, and in our experience produced considerably less pain than in cases for which we placed sutures circumferentially around the ribs – presumably due to the ability to avoid the neurovascular bundle during tying of the sutures.

Because of the large size of the defect, it was necessary to bridge the defect with a hernia prosthesis. Superficial skin ulcerations with draining sinus tracts were present, which led us to classify this as a grade III hernia according to the guidelines proposed in 2010 by the Ventral Hernia Working Group [9]. Because of the associated higher risk for subsequent infection and/or wound complications, we chose PADM (a biologic tissue matrix) over a synthetic mesh for implantation. This was a logical choice due to the superior resistance of biologic tissue matrices to infection in both a locally contaminated environment or in cases in which the matrix becomes exposed due to breakdown of the wound [6,10]. Surgical sites repaired with biologic tissue matrices heal by local tissue regeneration and revascularization rather than by scar formation. Once this revascularization occurs, it is proposed that there is no foreign-body response to the matrix, and that this reduces the rate of chronic infection and ulceration through the skin [6]. Additionally, biologic tissue matrices have been shown to revascularize despite the presence of bacterial contamination, and some matrices (PADM in particular) do not require explantation if infection occurs [10].

In this repair, we selected Strattice™, a non-crosslinked biologic tissue matrix derived from porcine dermis and processed physically, chemically, and enzymatically to remove cellular material and antigens. Our reason for selection was twofold. First, Strattice™ has demonstrated ability to revascularize and integrate into the recipient’s surrounding tissue. Strattice™ induces a fibroblastic reaction with focal tissue and capillary integration into the matrix that is consistent with a normal healing response [11]. In addition, biologic tissue matrices have been shown to be safe to use in the repair of contaminated ventral hernia defects [12]. These characteristics made Strattice™ an ideal choice for use in our patient due to the ulceration and infected overlying skin present prior to the time of surgery.

The success of biologic tissue matrices is thought to be due to rapid revascularization and tissue ingrowth, resulting in improved resistance to infection and more rapid integration into the host tissues. The use of omental flaps has been previously advocated as a means to provide vascularized tissue coverage for abdominal wall defects in combination with both synthetic mesh and biologic matrices [13,14]. The partial necrosis of the omental flap, which occurred in our patient nine weeks after the initial operation (believed to be due to torsion) necessitated reoperation and debridement, which allowed visualization of vigorous ongoing granulation of the implanted PADM, which we feel was facilitated by the coverage of the implant with the highly vascular omental tissue. The PADM was well incorporated at nine weeks postoperatively, which we also feel was facilitated by use of the omental coverage.

## Conclusions

In summary, we report a successful repair of a complicated subcostal hernia defect using a novel combination of techniques: a non-crosslinked PADM implant with a generous underlay, fixation to the rib superiorly using the Mitek™ bone anchor system, and coverage of the implant using a pedicled omental flap, which served to obliterate dead space, enhance the rate of revascularization, and reduce the chances of seroma formation. This case emphasizes the need for a thorough understanding of the challenges of the specific type of hernia defect encountered, as well as knowledge of available techniques that may be adjunctively employed to enhance the chances of achieving a successful result.

## Consent

Written informed consent was obtained from the patient for publication of this case report and any accompanying images. A copy of the written consent is available for review by the Editor-in-Chief of this journal and will be provided on request.

## Abbreviations

PADM: porcine-derived acellular dermal matrix.

## Competing interests

The authors received a grant from LifeCell Corporation, Branchburg, NJ, USA to cover manuscript preparation costs. Dr Richmond serves as a consultant for LifeCell Corporation. The opinions expressed in this article are those of the authors. The authors received no honoraria/fee for service related to the development of this article.

## Authors’ contributions

JK conducted the initial literature search and wrote the initial draft of the manuscript, as well as assisted in the performance of the operation in this case. JDH provided critical review and revision of the manuscript and was one of the two primary surgeons involved in the performance of the operation. BR was one of the two primary surgeons involved in the performance of the operation and provided critical review and revision of the manuscript. All authors approved the final version of the manuscript prior to submission.
